# Suboptimal Intake of Fruits and Vegetables in Nine Selected Countries of the World Health Organization European Region

**DOI:** 10.5888/pcd20.230159

**Published:** 2023-11-16

**Authors:** Holly L. Rippin, Katerina Maximova, Enrique Loyola, Joao Breda, Kremlin Wickramasinghe, Carina Ferreira-Borges, Nino Berdzuli, Morteza Hajihosseini, Irina Novik, Vital Pisaryk, Lela Sturua, Ainura Akmatova, Galina Obreja, Saodat Azimzoda Mustafo, Banu Ekinci, Toker Erguder, Shukhrat Shukurov, Gahraman Hagverdiyev, Diana Andreasyan, Sergei Bychkov, Ivo Rakovac

**Affiliations:** 1 World Health Organization European Office for the Prevention and Control of Non-Communicable Diseases, Division of Country Health Programmes, World Health Organization Regional Office for Europe, Copenhagen, Denmark; 2 MAP Centre for Urban Health Solutions, Li Ka Shing Knowledge Institute, St. Michael’s Hospital, Toronto, Ontario, Canada; 3 Dalla Lana School of Public Health, University of Toronto, Ontario, Canada; 4 Division of Country Health Policies and Systems, World Health Organization Greece, Athens, Greece; 5 World Health Organization Regional Office for Europe, Copenhagen, Denmark; 6 School of Public Health, University of Alberta, Edmonton, Alberta, Canada; 7 Republican Scientific and Practical Center of Medical Technologies, Informatization, Management and Economics of Public Health, Minsk, Belarus; 8 National Center for Disease Control and Public Health of Georgia, Tbilisi, Georgia; 9 Department of Public Health, Ministry of Health, Bishkek, Kyrgyzstan; 10 Department of Social Medicine and Management, Nicolae Testemitanu State University of Medicine and Pharmacy, Chisinau, Republic of Moldova; 11 State Research Institute of Gastroenterology, Ministry of Health and Social Protection of Population, Dushanbe, Republic of Tajikistan; 12 Department of Chronic Disease and Elderly Health, General Directorate of Public Health of Ministry of Health of Turkey, Ankara, Turkey; 13 World Health Organization Country Office in Turkey, Ankara, Turkey; 14 Central Project Implementation Bureau of the Health-3 Project, Tashkent, Uzbekistan; 15 Public Health and Reforms Center, Ministry of Health, Baku, Azerbaijan; 16 National Institute of Health, Ministry of Health, Yerevan, Armenia

## Abstract

The objective of this study was to characterize fruit and vegetable consumption in 9 selected countries of the World Health Organization (WHO) European Region. We analyzed data on fruit and vegetable intake and participant sociodemographic characteristics for 30,455 adults in 9 Eastern European and Central Asian countries via standardized STEPS survey methodology. Fruit and vegetable consumption across all countries was suboptimal, with a high percentage of populations not meeting the WHO-recommended intake of at least 5 servings (400 g) per day. Strengthened implementation of evidence-based policies to increase intake of fruit and vegetables is needed to reduce the burden of and disparities in NCDs.

**Summary**

**What is already known on this topic?**

Low rates of fruit and vegetable intake are associated with increased risk of noncommunicable diseases (NCDs). Although the disease burden due to inadequate fruit and vegetable consumption appears highest in Eastern Europe and Central Asia among countries in the World Health Organization (WHO) European Region, little systematic evidence exists.

**What is added by this report?**

Higher NCD death rates in Eastern Europe and Central Asia may be partly explained by differences in diet quality, particularly low rates of fruit and vegetable intake. Most populations in our study did not meet the WHO-recommended daily intake of at least 5 servings (400 g).

**What are the implications for public health practice?**

Evidence-based policies are needed to increase fruit and vegetable consumption and reduce the burden of and disparities in NCDs. Our findings can inform further research and policy development.

## Objective

Noncommunicable diseases (NCDs) account for 74% of deaths globally (1). Of all World Health Organization (WHO) regions, the European Region has the highest rates of NCD-related illness and death; almost 90% of deaths in this region are related to NCDs (2). Overweight and obesity affect more than 59% of adults in the European Region (2,3). Surveillance and monitoring are key to preventing and controlling NCDs (1). The WHO STEPwise approach to Surveillance (STEPS) is a standardized tool for collecting, analyzing, and disseminating data on NCD risk factors to guide and inform NCD policy makers on prevention policies (4).

WHO recommends daily consumption of at least 400 g (equivalent to 5 servings) of fruit and vegetables (5). Low consumption rates are associated with increased NCD risk (6). Increasing fruit and vegetable intake would, therefore, help achieve healthier diets and improve NCD outcomes (7,8).

Although the disease burden due to inadequate fruit and vegetable consumption appears highest in Eastern Europe and Central Asia among countries in the WHO European Region, little systematic evidence is available (9). Using STEPS data, we assessed fruit and vegetable consumption in 9 Eastern European and Central Asian countries. This evidence will help provide information for evidence-based policies to increase fruit and vegetable intake and reduce the effect of NCDs.

## Methods

The WHO STEPS surveyed 37,311 adults in Armenia, Azerbaijan, Belarus, Georgia, Kyrgyzstan, the Republic of Moldova, Tajikistan, Turkey, and Uzbekistan. The survey used a multistage clustered sampling design to collect population-based, cross-sectional, nationally representative household survey data from 2013 through 2018. Sampling procedures are detailed elsewhere (10,11). Informed consent was obtained by using country-specific language forms; ethical approval was obtained in each country before survey administration.

Face-to-face interviews and a standardized questionnaire assessed sociodemographic characteristics and NCD risk factors (11). Participants reported their age, sex, education level, marital status, and work status. Participants used visual aids to record the number of days per typical week and number of servings on each of those days that they consumed fruits and vegetables, from which the daily number of 80g servings was derived. Participants reported (yes/no) whether they received advice from a health care professional in the previous 3 years to eat at least 5 daily servings of fruits or vegetables. Trained interviewers measured height and weight at the participant’s home after the interview.

To enable comparisons across countries, we restricted our sample to adults aged 25 to 65 years. We considered participants who reported consuming 20 or more daily servings of fruit or vegetables to be outliers and excluded them from analyses. Our analytic sample size consisted of 30,455 participants. To estimate a nationally representative prevalence of fruit and vegetable consumption for each country, we calculated percentages derived in R version 3.5.0 *survey* package (R Foundation for Statistical Computing), which used survey design weights developed by WHO to account for multistage cluster design and nonresponse while considering the population age and sex distribution. We assessed differences in these percentages by using weighted multinomial mixed-effects regression adjusted for age, sex, marital status, and weight status (underweight, normal weight, overweight, obese), in *lme4* and *broom* packages in R version 3.5.0 (R Foundation for Statistical Computing). Analyses were stratified by country to facilitate comparisons and acknowledge country-specific contexts and cultural factors related to food intake. Significance was set at *P* < .05.

## Results

The average age of the study population was 42 years, and most participants were married or cohabiting (Table 1). In all countries, most participants had completed high school. Employment rates ranged from 37% to 80%. More than half were overweight or obese in all countries. The proportion of people not meeting the WHO recommendation to consume at least 5 daily servings of fruit or vegetables ranged from 60% in Tajikistan and 62% in Georgia to 88% in Turkey (Table 2). The average number of servings of fruit or vegetables per day was below the 5 recommended servings in all countries, except Tajikistan (5.1 servings/day).

**Table 1:** Characteristics of Selected Participants (N = 30,455) in WHO STEPS in 9 Countries in Eastern Europe and Central Asia, 2013–2017^a^

Characteristic	**Armenia (n = 1,878)**	**Azerbaijan (n = 4,700)**	**Belarus (n = 4,224)**	**Georgia (n = 3,399)**	**Kyrgyzstan (n = 2,623)**	**Republic of Moldova (n = 3,983)**	**Tajikistan (n = 2,237)**	**Turkey (n = 4,208)**	**Uzbekistan (n = 3,203)**
**Survey year**	2016	2017	2016	2016	2013	2013	2016	2017	2014
**Response rate, %**	82	97	87	76	100	84	99	70	89
**Mean age, y**	42	42	42	42	42	42	42	42	42
**Female, %**	47	51	51	52	50	49	45	51	49
**Marital status, %**									
Never married	12	10	15	15	5	8	3	12	5
Currently married or cohabiting	80	82	63	77	80	77	92	83	81
Other	8	8	22	8	15	15	5	5	14
**Highest level of education, %^b^**									
Less than high school	46	12	18	20	10	20	21	35	48
High school	28	43	53	22	66	58	63	46	39
College/university/postgraduate	25	44	28	58	23	23	16	19	13
**Work status, %**									
Employed	45	50	80	44	49	59	37	46	46
Nonpaid/homemaker/student	28	26	5	0.2	29	18	34	39	25
Retired	3	6	9	56	12	9	7	10	13
Unemployed	24	18	5	—c	10	14	23	5	15
**Weight status, %^d^**									
Underweight	5	4	2	3	2	2	2	0.9	2
Normal weight	43	34	37	31	39	36	36	27	36
Overweight	30	39	36	33	34	35	40	40	34
Obese	22	23	25	33	25	27	22	32	28

Abbreviation: WHO, World Health Organization.

a The WHO STEPwise approach to Surveillance (STEPS) is a standardized tool for collecting, analyzing, and disseminating data on NCD risk factors to inform NCD prevention policies (4); 37,311 adults participated in this survey during 2013–2018 in these 9 countries in WHO’s European Region; after exclusions, the analytic sample consisted of 30,455 adults aged 25 to 65 years. Percentages may not add to 100 because of rounding.

b Determined by using national education categories mapped to UNESCO’s (United Nations Educational, Scientific and Cultural Organization’s) International Standard Classification of Education (ISCED) (12). ISCED provides a comprehensive framework of uniform and internationally agreed definitions to facilitate comparisons of education systems across countries.

c Data not available.

d Weight status based on body mass index (BMI), derived from measured height and weight and calculated as weight in kg divided by height in m^2^: underweight, BMI <18.5, normal weight; BMI 18.5 to <25.0; overweight, BMI 25.0 to <30.0; obese, BMI ≥30.0.

**Table 2:** Fruit and Vegetable Consumption Among Selected Participants (N = 30,455) in WHO STEPS in 9 Countries in Eastern Europe and Central Asia, 2013–2017^a^

Measure	Armenia	Azerbaijan	Belarus	Georgia	Kyrgyzstan	Republic of Moldova	Tajikistan	Turkey	Uzbekistan
Mean no. of days fruit consumed/week	5.4	5.1	5.1	5.3	4.9	5.6	4.9	4.6	4.4
Mean no. of days vegetables consumed/week	5.0	5.9	5.6	6.0	5.3	5.9	6.6	5.1	6.2
Mean no. of servings of fruit consumed/day	2.2	2.1	2.2	2.5	2.3	2.3	2.5	2.0	2.4
Mean no. of servings of vegetables consumed/day	2.1	2.2	2.3	2.7	2.0	2.3	3.3	2.0	3.2
Mean no. of servings fruits and vegetables consumed/day	3.6	3.5	3.7	4.5	3.4	4.1	5.1	3.1	4.6
% Consuming <5 portions fruit and vegetables/day	76	76	73	62	74	65	60	88	67

Abbreviation: WHO, World Health Organization.

a The WHO STEPwise approach to Surveillance (STEPS) is a standardized tool for collecting, analyzing, and disseminating data on non-communicable disease (NCD) risk factors to inform NCD prevention policies (4); 37,311 adults participated in this survey during 2013–2018 in these 9 countries in WHO’s European Region; after exclusions, the analytic sample consisted of 30,455 adults aged 25 to 65 years.

Fruit and vegetable intake varied substantially by education, particularly in Armenia, Azerbaijan, Belarus, Kyrgyzstan, Republic of Moldova, and Tajikistan. Broadly, participants with more than a high school education consumed more servings of fruit and vegetables daily (Figure).

**Figure:**
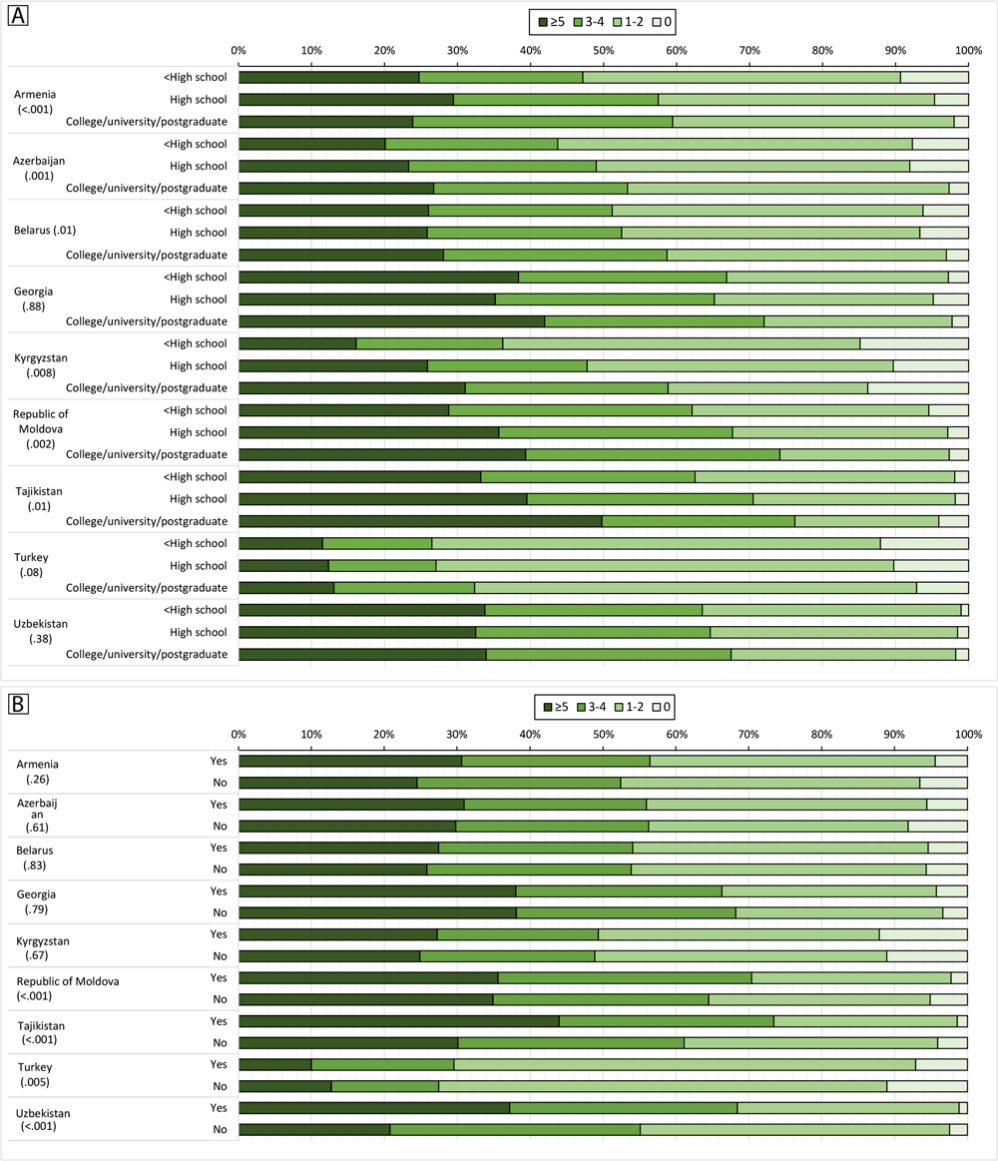
National prevalence of daily servings of fruit and vegetables (0, 1-2, 3-4, ≥5), by education (A) and by receipt of advice from a health care professional to eat at least 5 daily servings of fruits or vegetables (B). Education level was determined by using national education categories mapped to UNESCO’s (United Nations Educational, Scientific and Cultural Organization’s) International Standard Classification of Education (ISCED) (12). ISCED provides a comprehensive framework of uniform and internationally agreed definitions to facilitate comparisons of education systems across countries. Value in parentheses after country name is *P* value.

**Table:** 

A. By education						
Country	Groups	Daily servings of fruits and vegetables				*P* for difference
		0	1–2	3–4	≥5	
Armenia	Less than high school	9.3	43.5	22.4	24.7	<.001
	High school	4.7	37.8	28.1	29.4	
	College/university/postgraduate	2.0	38.6	35.6	23.9	
Azerbaijan	Less than high school	7.7	48.6	23.6	20.1	<.001
	High school	8.0	43.0	25.7	23.3	
	College/university/postgraduate	2.7	44.0	26.6	26.7	
Belarus	Less than high school	6.2	42.6	25.2	26.0	.02
	High school	6.7	40.9	26.6	25.9	
	College/university/postgraduate	3.0	38.3	30.6	28.1	
Georgia	Less than high school	2.8	30.4	28.5	38.4	.88
	High school	4.8	30.0	30.0	35.2	
	College/university/postgraduate	2.3	25.8	30.1	41.9	
Kyrgyzstan	Less than high school	14.9	48.9	20.1	16.1	.008
	High school	10.3	41.9	21.9	25.9	
	College/university/postgraduate	13.8	27.4	27.8	31.1	
Republic of Moldova	Less than high school	5.4	32.4	33.3	28.8	.002
	High school	2.8	29.5	32.0	35.7	
	College/university/postgraduate	2.6	23.2	34.8	39.3	
Tajikistan	Less than high school	1.9	35.6	29.3	33.2	.01
	High school	1.8	27.7	31.0	39.5	
	College/university/postgraduate	4.1	19.7	26.4	49.8	
Turkey	Less than high school	12.1	61.4	15.0	11.5	.08
	High school	10.2	62.7	14.7	12.4	
	College/university/postgraduate	7.1	60.6	19.3	13.1	
Uzbekistan	Less than high school	1.0	35.5	29.8	33.8	.38
	High school	1.5	33.9	32.1	32.5	
	College/university/postgraduate	1.8	30.8	33.6	33.9	
**B. By receipt of advice from health care professional**						
Armenia	Yes	4.4	39.2	25.8	30.6	.26
	No	6.5	41.0	27.9	24.5	
Azerbaijan	Yes	5.6	38.5	25.0	31.0	.61
	No	8.1	35.6	26.5	29.8	
Belarus	Yes	5.4	40.5	26.6	27.4	.83
	No	5.7	40.5	28.1	25.8	
Georgia	Yes	4.3	29.4	28.3	38.1	.79
	No	3.4	28.4	30.1	38.1	
Kyrgyzstan	Yes	12.1	38.5	22.1	27.3	.67
	No	11.1	40.1	24.0	24.9	
Republic of Moldova	Yes	2.2	27.4	34.8	35.6	<.001
	No	5.1	30.4	29.6	34.9	
Tajikistan	Yes	1.4	25.2	29.4	44.0	<.001
	No	4.1	34.8	31.0	30.1	
Turkey	Yes	7.1	63.3	19.6	10.0	.005
	No	11.0	61.5	14.7	12.7	
Turkmenistan	Yes	1.3	8.4	15.1	75.2	.80
	No	0.0	7.7	10.0	82.3	
Uzbekistan	Yes	1.1	30.4	31.3	37.2	<.001
	No	2.5	42.4	34.3	20.8	

Rates of fruit and vegetable consumption were higher among participants who had received advice from a health care professional to eat at least 5 daily servings of fruits or vegetables, particularly in the Republic of Moldova, Tajikistan, Turkey, and Uzbekistan (Figure). Rates of fruit and vegetable consumption were also higher among those who were overweight or obese, older participants, and among women.

Although fruit and vegetable consumption varied by work status, particularly in Armenia, Azerbaijan, Republic of Moldova, and Tajikistan, we found no clear pattern between or within countries. Similarly, consumption varied by marital status, particularly in the Republic of Moldova and Tajikistan, but no clear pattern emerged.

## Discussion

This study used WHO STEPS data to assess fruit and vegetable consumption in 9 Eastern European and Central Asian countries in the WHO European Region. National consumption varied, but no country met WHO’s recommendation of at least 5 servings (400 g) per day, except for Tajikistan (5.1 servings/day). Participants with more education generally consumed more daily servings, mirroring regional trends (13) and suggesting that education interventions could improve fruit and vegetable intake and, therefore, population health. Availability, affordability, and national income and development level may influence this complex relationship. Further research is needed into the relationship between fruit and vegetable consumption and education, availability, and affordability.

In some countries, participants receiving advice from health care professionals to consume at least 5 daily servings of fruit or vegetables had higher intakes than participants not receiving this advice. Health care provider–patient consultation time could be used more effectively to improve fruit and vegetable intake; for example, brief interventions are a WHO “Best Buys” intervention (14). A suite of policy options and public health strategies, such as procurement policies, in-store promotions, and subsidies, is needed to increase population-level fruit and vegetable consumption (14,15). More research on the relationship between those receiving advice and fruit and vegetable intake would help prioritize policy development.

Our study has strengths and limitations. The STEPS survey has an extensive infrastructure and a standardized methodology. Our study is the first to systematically assess fruit and vegetable consumption by using comparable indicators in Eastern European and Central Asian countries in the WHO European Region. The data are nationally representative with a high response rate, but the survey design is cross-sectional, which precludes causal inference. The data are self-reported, so they rely on participants’ understanding and accurate reporting of their fruit and vegetable intake. The data are also dated (2013–2017) and do not show trends over time.

Higher NCD-related death rates in Eastern Europe and Central Asia may be partly explained by differences in diet quality, particularly rates of low fruit and vegetable consumption. Our study found that fruit and vegetable consumption in all countries was suboptimal. Survey participants with higher education who had received advice from a health care professional to eat at least 5 daily servings of fruit or vegetables generally consumed more fruits and vegetables in some countries. Awareness of the 5-a-day recommendation and the ability to operationalize awareness could lead to higher intakes, possibly especially in populations that are overweight. Evidence-based policies are needed to increase fruit and vegetable consumption and reduce the burden of and disparities in NCDs. Policy makers can use our findings to initiate further research and policy development.
